# A common *p73* polymorphism is associated with a reduced incidence of oesophageal carcinoma

**DOI:** 10.1054/bjoc.2001.2066

**Published:** 2001-11

**Authors:** B M Ryan, R McManus, J S Daly, E Carton, P W N Keeling, J V Reynolds, D Kelleher

**Affiliations:** Departments of Clinical Medicine and Gastroenterology, Surgery, St. James's Hospital and Trinity College, Dublin 8, Ireland

**Keywords:** oesophageal cancer, p53, p73, polymorphism, genetics, risk

## Abstract

The incidence of oesophageal adenocarcinoma is rising; to date, no susceptibility genes have been identified. p73, a novel p53 homologue, maps to chromosome 1p36, a region commonly deleted in oesophageal cancers. p73 shares some p53-like activity, but in addition, may also play a role in gastrointestinal epithelial inflammatory responses. A non-coding *p73* polymorphism (denoted AT or GC) may be functionally significant. We investigated whether this polymorphism might play a role in the aetiopathogenesis of oesophageal cancer. This was a case–control, retrospective study. 84 cases of oesophageal cancer (25 squamous and 59 adenocarcinoma) and 152 normal population controls were genotyped for this polymorphism. Informative cases were examined for *p73* LOH within the tumour. AT/AT homozygotes were significantly less prevalent in the oesophageal cancer population (1/84 = 1.2%) compared to controls (15/152 = 9.9%) (*P* < 0.02), corresponding to an odds ratio of 0.11 (95% C.I. 0.02–0.6, *P* < 0.02), or 9-fold reduced risk. Moreover, AT/AT homozygotes were significantly less frequent in the cancer population than would be expected under the Hardy–Weinberg hypothesis (*P* = 0.0099). LOH at the *p73* locus was observed in 37.8% (14/37) of the AT/GC heterozygotes studied; in all cases there was loss of the AT allele. Our findings indicate that *p73* AT/AT homozygotes appear to be protected against the development of oesophageal cancer. Clinically, this observation could have implications in aiding identification of high-risk Barrett's oesophagus patients.© 2001 Cancer Research Campaign  http://www.bjcancer.com


*British Journal of Cancer* (2001) **85**(10), 1499–1503
© 2001 Cancer Research Campaign
doi: 10.1054/ bjoc.2001.2066, available online at http://www.idealibrary.com on IDEAL
http://www.bjcancer.com

### A common *p73* polymorphism is associated with a reduced incidence of oesophageal carcinoma

**BM Ryan[1], R McManus[1], JS Daly[1], E Carton[2], PWN Keeling[1], JV Reynolds[2] and D Kelleher[1]**

Departments of [1]Clinical Medicine and Gastroenterology and [2]Surgery, St. James's Hospital and Trinity College, Dublin 8, Ireland

**Summary** The incidence of oesophageal adenocarcinoma is rising; to date, no susceptibility genes have been identified. p73, a novel p53 homologue, maps to chromosome 1p36, a region commonly deleted in oesophageal cancers. p73 shares some p53-like activity, but in addition, may also play a role in gastrointestinal epithelial inflammatory responses. A non-coding *p73* polymorphism (denoted AT or GC) may be functionally significant. We investigated whether this polymorphism might play a role in the aetiopathogenesis of oesophageal cancer. This was a case–control, retrospective study. 84 cases of oesophageal cancer (25 squamous and 59 adenocarcinoma) and 152 normal population controls were genotyped for this polymorphism. Informative cases were examined for *p73* LOH within the tumour. AT/AT homozygotes were significantly less prevalent in the oesophageal cancer population (1/84 = 1.2%) compared to controls (15/152 = 9.9%) ($P < 0.02$), corresponding to an odds ratio of 0.11 (95% C.I. 0.02–0.6, $P < 0.02$), or 9-fold reduced risk. Moreover, AT/AT homozygotes were significantly less frequent in the cancer population than would be expected under the Hardy–Weinberg hypothesis ($P = 0.0099$). LOH at the *p73* locus was observed in 37.8% (14/37) of the AT/GC heterozygotes studied; in all cases there was loss of the AT allele. Our findings indicate that *p73* AT/AT homozygotes appear to be protected against the development of oesophageal cancer. Clinically, this observation could have implications in aiding identification of high-risk Barrett's oesophagus patients. © 2001 Cancer Research Campaign http://www.bjcancer.com

**Keywords**: oesophageal cancer; p53; p73; polymorphism; genetics; risk

Oesophageal cancer is a highly malignant disease associated with a dismal prognosis (Walsh et al, 1996; Bosset et al, 1997). The incidence of oesophageal adenocarcinoma has risen 6- to 8-fold in Western Europe and in the US over the past 3 decades (Cohen and Parkman, 1999). By contrast, the incidence of oesophageal squamaous carcinoma has remained static. It has been recognised that Barrett's oesophagus, a result of chronic gastro-oesophageal reflux, is the key intermediary step in the development of oesophageal adenocarcinoma. A recent report however, has suggested that chronic symptomatic gastro-oesophageal reflux, even in the absence of Barrett's oesophagus, may be an independent risk factor for the development of oesophageal adenocarcinoma (Lagergren et al, 1999). Therefore, identification of factors which modulate the risk of development of oesophageal cancer in patients with reflux oesophagitis or Barrett's oesophagus, could prove of great value in the future, in structuring screening programmes.

To date, no susceptibility genes for oesophageal adenocarcinoma or squamous cancer have been identified. The molecular biology of oesophageal cancer is poorly defined (Montesano et al, 1996). Tumours are often described at an advanced stage, by which time they have accumulated a diversity of mutations and deletions. In contrast with other tumour types however, *p53* mutation appears to be an early event in oesophageal carcinogenesis (Montesano et al, 1996; Barrett et al, 1999). A recent study has indicated that the evolution of neoplastic cell lineages in Barrett's oesophagus and oesophageal adenocarcinoma is complex, involving non-random loss of heterozygosity (LOH), duplications and mutations which do not occur in a particular obligate order (Barrett et al, 1999).

Recently, p73, a novel structural and functional homologue of p53 was identified (Kaghad et al, 1997). *p73* maps to chromosome 1p36 (Kaghad et al, 1997), a region deleted in up to 40% of oesophageal cancers (Barrett et al, 1996). *p73* is a putative imprinted tumour suppressor gene, which has been shown to be imprinted in some, but not all human tissues investigated to date (Kaghad et al, 1997; Mai et al, 1998a, 1998b; Nomoto et al, 1998; Yokomizo et al, 1999). Loss of heterozygosity (LOH) at the *p73* locus has been reported with varying frequency in different tumours; 5.3% of prostatic cancers (Takahashi et al, 1998), 19% of neuroblastomas (Ichimiya et al, 1999), 42% of lung cancers (Nomoto et al, 1998) and 8% (Nimura et al, 1998) and 64% (Cai et al, 2000) of oesophageal cancers have reported *p73* LOH. However, extensive mutational analysis of *p73* in diverse tumours has demonstrated that mutations in this gene are rare, being reported in fewer than 2% of all cancers screened (Mai et al, 1998b; Takahashi et al, 1998; Yokomizo et al, 1999), suggesting that this gene does not function as a traditional tumour-suppressor gene. Two linked, single nucleotide polymorphisms, forming a compound polymorphism at positions 4 ($G \rightarrow A$) and 14 ($C \rightarrow T$) have been identified in the 5′ untranslated region (UTR), of exon 2 of the *p73* gene (Kaghad et al, 1997), and have been designated the AT and GC alleles respectively. This polymorphism lies just upstream of the initiating AUG of exon 2, in a region which may theoretically form a stem-loop structure. It has been suggested that this could potentially affect gene expression, perhaps through alteration of the efficiency of translation initiation (Kaghad et al, 1997). Recently, a p73-knockout mouse model has been developed; these mice manifested various neurohormonal deficits and also inflammatory defects, in particular, the mice exhibited severe

*Received 11 May 2001*
*Revised 16 July 2001*
*Accepted 23 July 2001*

*Correspondence to:* B Ryan haemorrhagic inflammation and erosion of the gastrointestinal tract (Yang et al, 2000).

In this study, we sought to investigate whether the AT/GC polymorphism in the *p73* gene may play a role in the pathogenesis of oesophageal cancer. To this end, we analysed the *p73* genotype of 84 oesophageal cancer cases and compared with that of 152 healthy normal population controls. Furthermore, we investigated the frequency of *p73* LOH in informative cases.

### MATERIALS AND METHODS

### Cases and controls

152 healthy age- and sex-matched normal population controls were chosen from a databank of controls stored at our institution. This databank contains over 300 whole blood samples from both healthy individuals working at the hospital, and healthy elderly individuals in the community, with an age range from 21–86 years. DNA was extracted from whole blood. Ethical approval was obtained from our institution's Ethics Committee.

84 cases of oesophageal cancer, diagnosed between 1997 and 1999, and for whom tissue samples were readily available, were identified from the hospital oesophageal cancer database (25 squamous carcinomas and 59 adenocarcinomas) which contains over 400 cases of oesophageal carcinoma diagnosed since 1990 (Walsh et al, 1996). Normal genomic DNA was extracted either from a sample of normal frozen or paraffin-embedded tissue (57 cases), or from a whole blood sample where available (27 cases). Tumour DNA was extracted either from frozen or paraffin-embedded tissue. All frozen tissue samples were snap frozen in liquid nitrogen, and stored at –80˚C until analysed. Two oesophageal cell lines were also examined, a squamous carcinoma (ECACC ref. # OE21) and an adenocarcinoma cell line (ECACC ref. # OE 19).

### DNA isolation

Tumour DNA was isolated from 50–100 mg of frozen tissue sample using the Genosys RNA Isolator® kit, or from paraffin-embedded specimens using a standard phenol/chloroform precipitation protocol. DNA was isolated from whole blood samples using Qiagen® blood mini-kits.

### Genotyping and examination of LOH at the *p73* locus

The polymorphic region of exon 2 of the *p73* gene was amplified from genomic DNA extracted from both normal and tumour tissues, using previously described primers (Yokomizo et al, 1999). The PCR product was then digested with *Sty* I (New England Biolabs). The presence of the AT polymorphism creates a *Sty* I digestion site, yielding products of 157 bp and 72 bp; the GC allele remains uncleaved by *Sty* I, yielding a 229 bp product. The products were then resolved by electrophoresis on 2% agarose (Figure 1). In all experiments, an AT/AT homozygote was included, to act as a control for complete digestion of the PCR products by the *Sty* I enzyme.

### Statistical analysis

Patient and control populations were compared for differences in genotype frequencies using a $\chi^2$ test (SAS, statistical analysis software). Divergences in allele distribution within populations, from those predicted under Hardy–Weinberg equilibrium, were tested for significance using GENEPOP software, version 3.1b (Raymond and Rousset, 1995).

### RESULTS

### Genotypic analysis of cases and controls

Genetic analysis of the patient and control populations demonstrated that the AT/AT genotype was significantly less prevalent in the cancer group compared to the normal population (1.2% v 9.9%, $P < 0.02$) (Table 1). Furthermore, as genotype frequencies within a given population are inextricably linked, both populations were concomitantly examined for differences between all genotype frequencies, giving a total $\chi^2$ across groups of 6.527 (2 degrees of freedom, $P = 0.038$); the vast bulk of which was attributable to the difference between AT/AT homozygotes. The AT homozygote was only detected in 1/25 squamous carcinomas and 0/59 adenocarcinomas (Table 1). The odds ratio (OR) for development of oesophageal cancer in AT/AT homozygotes compared to all other genotypes was 0.11 (95% CI = 0.02–0.6), corresponding to a 9-fold reduced risk in these individuals. Despite the marked differences in allele distribution, the overall AT allele frequency was only slightly lower in the cancer (0.2559) compared to the control (0.3125) population (*P* = n.s.); reduced numbers of homozygotes are compensated by an excess of AT/GC heterozygotes in the patient population (Table 1).

Hardy–Weinberg equilibrium analysis of allele distribution within our cancer and control populations was performed (Table 2). The allele distribution in the cancer population deviated significantly ($P = 0.0099$, GENEPOP (Raymond and Rousset, 1995)), from that expected. In the cancer population the prevalence of AT/AT (and also GC/GC) homozygotes was lower than expected,

**Table 1**: Demographics and *p73* genotype frequencies in the control and oesophageal cancer populations

| | | | | GENOTYPE (*n* %) | | | | |
|---|---|---|---|---|---|---|---|---|
| | ***n*** | **Median age (y)** | **Age range (y)** | **AT/AT** | **AT/GC** | **GC/GC** | **AT allele frequency** | ***P* value** |
| Controls | 152 | 58 | 43–86 | 15 (9.9)* | 65 (42.8) | 72 (47.3) | 0.3125¶ | *P < 0.02<br>*OR 0.11 (0.02–0.6) |
| Oesophageal Cancer Cases | 84 | 73 | 53–77 | 1 (1.2)* | 41 (48.8) | 42 (50) | 0.2559¶ | *P* = ns |
| *Adenocarcinomas* | 59 | 72.5 | 48–72 | 0 (0) | 29 (49.1) | 30 (50.8) | 0.2458 | |
| *Squamous carcinomas* | 25 | 74 | 53–77 | 1 (4) | 12 (48) | 12 (48) | 0.2800 | |

*AT/ATs were significantly less frequent in the cancer population, with an odds ratio (OR) for development of oesophageal cancer in AT/ATs of 0.11 (95% CI 0.02–0.6). This was significant also when adenocarcinoma cases were analysed separately, but not when squamous cancers were analysed separately, due to the small numbers in this group. ¶There was no significant difference in AT allele frequency between the cases and controls.

 © 2001 Cancer Research Campaign

**Table 2** Hardy–Weinberg analysis of allele distribution in controls and oesophageal cancer cases

| | Genotype *(n)* | | | | | | *P* value |
|---|---|---|---|---|---|---|---|
| | AT/AT | | AT/GC | | GC/GC | | |
| | Observed | Expected | Observed | Expected | Observed | Expected | |
| Controls* | 15 | 14.7 | 65 | 65.5 | 72 | 71.7 | **P* = ns |
| Oesophageal cancer cases§ | 1 | 5.4 | 41 | 32.2 | 42 | 46.4 | §*P* = 0.009 |

*The observed genotype frequency and allele distribution in the control population did not differ significantly from that expected under Hardy–Weinberg equilibrium (HWE). §The observed genotype frequency and allele distribution in the oesophageal cancer population was significantly different from that expected under HWE.

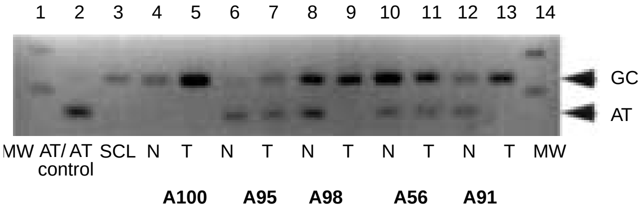

**Figure 1** *p73* genotyping and LOH in oesophageal cancer cases. This shows a representative sample of results of genotyping of matched normal (N) and tumour (T) samples. Lanes 1 and 14: Molecular weight marker (MW). Lane 2 shows an AT/AT homozygote, which was used in each experiment as an internal control of complete *Sty 1* digestion. Lane 3, squamous carcinoma cell line OE21, showing presence of GC allele only. Lanes 4 & 5: Matched tissues of case A100, a GC homozygote. Lanes 6 & 7: Matched tissues from case A95 a heterozygote, demonstrating heterozygosity in N and T tissues. Lanes 8 & 9: Case A98, showing LOH of the AT allele in the tumour tissue. Lanes 10 & 11: Case A56, a heterozygote. Lanes 12 & 13: Case A 91, showing LOH of the AT allele in the T tissue whereas there was heterozygote excess (Table 2). In contrast, the allele distribution in the control population was almost exactly as predicted (Table 2).

### Loss of heterozygosity in informative cases

Tumour tissue was available for 37/41 heterozygote cases. *p73* LOH was demonstrated in 14/37 cases (37.8%). In all instances of *p73* LOH, we observed exclusive loss of the AT allele (Figure 1; lanes 8 & 9 and lanes 12 & 13). We also studied two oesophageal carcinoma cell lines, ECACC #OE21 (squamous carcinoma) and ECACC #OE19 (adenocarcioma), both of which were GC homo/hemi-zygotes.

### DISCUSSION

We found a significant association between the *p73* AT/GC polymorphism and the risk of developing oesophageal cancer. AT/AT homozygotes in our study were significantly less prevalent in the oesophageal cancer population, both in adenocarcinomas (0/59) and squamous carcinomas (1/25), compared to the control population, OR 0.11 (95% CI 0.02–0.6, $P < 0.02$). Adenocarcinomas and squamous carcinomas were analysed together, because of the smaller numbers in each separate group. AT/AT homozygotes were significantly less frequent than expected in the cancer population, given the frequency of the AT allele in this group ($P = 0.0099$). Deviations from Hardy–Weinberg equilibrium such as those observed in this study, are strongly suggestive of an underlying selective process, which in this instance, appears to militate against the development of oesophageal cancer in AT/AT homozygotes. Population admixture may cause artefactual associations between allelic variants and disease risk (Altshuler et al, 1998), however, there is little or no population admixture in our study population (Irish), and both our control and cancer groups were derived from this homogenous population.

In this study, LOH of the *p73* locus was observed in 37.8% of informative (AT/GC heterozygote) oesophageal cancer cases. Importantly, there was exclusive loss of the AT allele in all instances of LOH, again, suggesting a specific functional role for the AT allele. A number of previous studies which have identified LOH at the *p73* locus in various human cancers, have not reported exclusive deletion of the AT allele (Nomoto et al, 1998; Takahashi et al, 1998; Ichimiya et al, 1999). However, in the landmark *p73* study, which examined tumour cell lines from a variety of origins, only 3/19 (16%) had the AT allele present at genomic level (Kaghad et al, 1997), the remainder being GC homo/hemi-zygotes. This suggests that loss of the *p73* AT allele may also occur in cancers of other tissue origins. Two previous studies have investigated LOH at the *p73* locus in oesophageal cancer patients of Asian ethnic background, and have reported vastly differing frequencies of LOH, ranging from 8% (2/25) (Nimura et al, 1998) to 64% (9/14) (Cai et al, 2000). In the most recent report, which found LOH in 64% of all informative cases, in 82% of instances, there was loss of the AT allele (Cai et al, 2000), similar to that observed in our current study.

In contrast with other tumour types, *p53* mutation is an early event in oesophageal carcinogenesis (Montesano et al, 1996; Barrett et al, 1999). P53 is a critical regulator of the cell cycle (Levine, 1997) and p73 shares significant functional homology with p53 (Kaghad et al, 1997; Zhu et al, 1998). Existing evidence shows that *p73* plays a role in the regulation of the cell cycle, apoptosis and possibly control of differentiation (Jost et al, 1997; Kaghad et al, 1997; DeLaurenzi et al, 1998; Zhu et al, 1998; Gong et al, 1999), all of which are critical in the oncogenic process. These existing data suggest that p73 activity may be capable of compensating, to some extent, for the early loss of p53 function (through mutation) observed in pre-cancerous oesophageal lesions (Barrett et al, 1999). Indeed, supporting this hypothesis, is the observation that increased expression of *p73* was found in oesophageal squamous cancers compared to matched normal tissues; moreover, increased *p73* expression significantly correlated with the presence of a p53 defect (Cai et al, 2000). Furthermore, increased *p73* expression occurred even in instances of LOH, suggesting that the level of expression is independent of LOH. Increased *p73* expression in tumour tissue relative to matched normal tissue, has also been observed in oesophageal adenocarcinomas, in preliminary work by our group (BR, unpublished data). The over-expression of *p73* may be a partial compensatory mechanism for the loss of p53 function that is commonly

© 2001 Cancer Research Campaign 

observed in oesophageal cancers, though obviously this is not sufficient to prevent the development of cancer. Conflicting reports show predominant mono-allelic (Kaghad et al, 1997; Mai et al, 1998b; Yokomizo et al, 1999) or bi-allelic *p73* expression in various normal tissues studied. We found predominant bi-allelic *p73* expression in normal and cancerous oesophageal tissues; mono-allelic expression of *p73* was observed in only 17% of normal tissues analysed (BR, unpublished data). Qualitative loss of the AT allele, despite a quantitative increase in overall *p73* expression, may be important in oesophageal epithelial function: Differential *p73* function or splice variant expression, perhaps related to this AT/GC polymorphism could potentially play a critical role in determining the development, or progression of early oesophageal lesions.

Recently it has been shown that p73 knockout mice develop severe gastrointestinal inflammation, suggesting that p73 may modulate inflammatory responses in the gastrointestinal tract mucosa (Yang et al, 2000). The p73-deficient mice were not observed to have an increased incidence of spontaneous tumours, however, the majority died early, within the first 30 days of life due to severe gastrointestinal haemorrhage (Yang et al, 2000). Chronic gastro-oesophageal reflux is known to predispose to the development of chronic oesophagitis with subsequent evolution of the Barrett's metaplasia-to-dysplasia-to-carcinoma sequence. In this context, the findings our study suggest that the *p73* AT allele might play a role in oesophageal mucosal regulation and in oesophageal carcinogenesis in a Caucasian population, perhaps through modulation of the inflammatory response to chronic gastro-oesophageal reflux.

The current report included 25 squamous carcinomas, and similar results were found to those in adenocarcinomas; while chronic oesophageal inflammation is a well-recognised precursor of oesophageal adenocarcinoma, the same does not apply for the pathogenesis of squamous carcinoma. However chronic irritation and inflammation of the oesophagus in response to other environmental factors such as alcohol, is also thought to play a role in the development of squamous carcinoma of the oesophagus.

The role of this AT/GC polymorphism in modulating p73 function requires further assessment. However, as the polymorphism is non-coding, unravelling the precise mechanisms through which it modulates p73 function, either qualitatively or quantitatively will be complex. Potential mechanisms include regulation of p73 expression at a transcriptional or translational level.

Clinically, genotyping for this polymorphism may lead to better identification of patients who are at increased risk of developing oesophageal cancer. This could prove of particular value in the management and surveillance of patients with Barrett's oesophagus, as intensive screening might be reserved for those with an increased genetic risk of developing oesophageal cancer. Indeed, given the recent observation that prolonged oesophageal reflux may constitute a risk factor for development of oesophageal cancer, even in the absence of Barrett's mucosa (Lagergren et al, 1999); then it may also prove necessary in the future, to identify patients with reflux oesophagitis, who are at risk of progression to oesophageal cancer.

### ACKNOWLEDGEMENTS

BM Ryan is supported by PPP Healthcare Medical Trust, UK. R McManus is a Wellcome Lecturer. JS Daly is supported by a grant from the Irish Health Research Board. We wish to thank Tony O'Grady for advice on statistical methods. This work was presented in abstract form at the Digestive Disease week, in San Diego, 2000.

### REFERENCES

Altshuler D, Kruglyak L and Lander E (1998) Genetic polymorphisms and disease (correspondance). *N Engl J Med* **338**: 1626

Barrett M, Sanchez C, Prevo L, Wong D, Galipeau P, Paulson T, Rabinovitch P and Reid B (1999) Evolution of neoplastic cell lineages in Barrett oesophagus. *Nature Genet* **22**: 106–109

Barrett MT, Galipeau PC, Sanchez CA, Emond MJ and Reid BJ (1996) Determination of the frequency of loss of heterozygosity in esophageal adenocarcinoma by cell sorting, whole genome amplification and microsatellite polymorphisms. *Oncogene* **12**: 1873–1878

Bosset J, Gignoux M, Triboulet J, Tiret E, Mantion G, Elias D, Lozach P, Ollier J, Pavy J, Mercier M and Sahmoud T (1997) Chemoradiotherapy followed by surgery compared with surgery alone in squamous cell cancer of the esophagus. *N Engl J Med* **337**: 161–167

Cai Y, Yang G.-Y, Nie Y, Wang L.-D, Zhao X, Song Y.-L, Seril D, Liao J, Xing E and Yang C (2000) Molecular alterations of *p73* in human esophageal squamous cell carcinomas: loss of heterozygosity occurs frequently; loss of imprinting and elevation of *p73* expression may be related to defective p53. *Carcinogenesis* **21**: 683–689

Cohen S and Parkman H (1999) Heartburn-A Serious Symptom (editorial). *N Engl J Med* **340**: 878–879

De Laurenzi V, Costanzo A, Barcaroli D, Terrinoni A, Falco M, Annicchiarico-Petruzzelli M, Levrero M and Melino G (1998) Two new p73 splice variants, g and d, with different transcriptional activity. *J Exp Med* **188**: 1763–1768

Gong J, Constanzo A, Yang HQ, Melino G, Kaelin WG, Levrero M and Wang JYJ (1999) The tyrosine-kinase c-Abl regulates p73 in apoptotic response to cisplatin-induced DNA damage. *Nature* **399**: 806–809

Ichimiya S, Nimura Y, Kageyama H, Takada N, Sunahara M, Shishikura T, Nakamura Y, Sakiyama S, Seki N, Ohira M, Kaneko Y, McKeon F, Caput D and Nakagawara A (1999) p73 at chromosome 1p36.3 is lost in advanced stage neuroblastoma but its mutation is infrequent. *Oncogene* **18**: 1061–1066

Jost CA, Marin MC and Kaelin WG (1997) p73 is a human p53- related protein that can induce apoptosis. *Nature* **389**: 191–194

Kaghad M, Bonnet H, Yang A, Creancier L, Biscan JC, Valent A, Minty A, Chalon P, Lellas JM, Dumont X, Ferrara P, McKeon F and Caput D (1997) Monoallelically expressed gene related to p53 at 1p36, a region frequently deleted in neuroblastoma and other human cancers. *Cell* **90**: 809–819

Lagergren J, Bergstrom R, Lindgren A and Nyren O (1999) Symptomatic gastroesophageal reflux as a risk factor for esophageal adenocarcinoma. *N Engl J Med* **340**: 825–831

Levine AJ (1997) P53, the cellular gatekeeper for growth and division (review). *Cell* **88**: 323–331

Mai M, Qian C, Yokomizo A, Tindall DJ, Bostwick D, Polychronakos C, Smith DI and Liu W (1998a) Loss of imprinting and allele switching in renal cell carcinoma. *Oncogene* **17**: 1739–1741

Mai M, Yokomizo A, Qian C, Yang P, Tindall DJ, Smith DI and Liu W (1998b) Activation of p73 silent allele in lung cancer. *Cancer Res* **58**: 2347–2349

Montesano R, Hollstein M and Hainaut P (1996) Genetic alterations in esophageal cancer and their relevance to etiology and pathogenesis: A review. *Int J Cancer* **69**: 225–235

Nimura Y, Mihara M, Ichimiya S, Sakiyama S, Seki N, Ohira M, Nomura N, Fujimori M, Adachi W, Amano J, He M, Ping YM and Nakagawara A (1998) P73, a gene related to p53, is not mutated in esophageal carcinomas. *Int J Cancer* **78**: 437–440

Nomoto S, Haruki N, Kondo M, Konishi H, Takahashi T, Takahashi T and Takahashi T (1998) Search for mutations and examination of allalic expression imbalance of the p73 gene at 1p36.33 in human lung cancers. *Cancer Res* **58**: 1380–1383

Raymond M and Rousset F (1995) GENEPOP (version 1.2): population genetics software for exact tests and ecumenicism. *J Heredity* **86**: 248–249

Takahashi H, Ichimiya S, Nimura Y, Watanabe M, Furusato M, Wakui S, Yatani R, Aizawa S and Makagawara A (1998) Mutation, allelotyping and transcription analyses of the p73 gene in prostatic carcinoma. *Cancer Res* **58**: 2076–2077

Walsh T, Noonan N, Hollywood D, Kelly A, Keeling N and Hennessy T (1996) A comparison of multimodal therapy and surgery for esophageal adenocarcinoma. *N Engl J Med* **335**: 462–467

Yang A, Walker N, Bronson R, Kaghad M, Oosterwegel M, Bonnin J, Vagner C, Bonnet H, Dikkes P, Sharpe A, McKeon F and Caput D (2000) p73-deficient

 *© 2001 Cancer Research Campaign*

mice have neurological, pheromonal and inflammatory defects but lack spontaneous tumours. *Nature* **404**: 99–103
Yokomizo A, Mai M, Tindall DJ, Cheng L, Bostwick DG, Naito S, Smith DI and Liu W (1999) Overexpression of the wild type p73 gene in human bladder cancer. *Oncogene* **18**: 1629–1633
Zhu J, Jiang J, Zhou W and Chen X (1998) The potential tumour suppressor p73 differentially regulates cellular p53 target genes. *Cancer res* **58**: 5061–5965

© 2001 Cancer Research Campaign

## References

[PDF_00365] Altshuler D., Kruglyak L., Lander E. (1998). Genetic polymorphisms and disease.. N Engl J Med.

[PDF_00370] Barrett M. T., Galipeau P. C., Sanchez C. A., Emond M. J., Reid B. J. (1996). Determination of the frequency of loss of heterozygosity in esophageal adenocarcinoma by cell sorting, whole genome amplification and microsatellite polymorphisms.. Oncogene.

[PDF_00367] Barrett M. T., Sanchez C. A., Prevo L. J., Wong D. J., Galipeau P. C., Paulson T. G., Rabinovitch P. S., Reid B. J. (1999). Evolution of neoplastic cell lineages in Barrett oesophagus.. Nat Genet.

[PDF_00374] Bosset J. F., Gignoux M., Triboulet J. P., Tiret E., Mantion G., Elias D., Lozach P., Ollier J. C., Pavy J. J., Mercier M. (1997). Chemoradiotherapy followed by surgery compared with surgery alone in squamous-cell cancer of the esophagus.. N Engl J Med.

[PDF_00378] Cai Y. C., Yang G. Y., Nie Y., Wang L. D., Zhao X., Song Y. L., Seril D. N., Liao J., Xing E. P., Yang C. S. (2000). Molecular alterations of p73 in human esophageal squamous cell carcinomas: loss of heterozygosity occurs frequently; loss of imprinting and elevation of p73 expression may be related to defective p53.. Carcinogenesis.

[PDF_00383] Cohen S., Parkman H. P. (1999). Heartburn--a serious symptom.. N Engl J Med.

[PDF_00386] De Laurenzi V., Costanzo A., Barcaroli D., Terrinoni A., Falco M., Annicchiarico-Petruzzelli M., Levrero M., Melino G. (1998). Two new p73 splice variants, gamma and delta, with different transcriptional activity.. J Exp Med.

[PDF_00388] Gong J. G., Costanzo A., Yang H. Q., Melino G., Kaelin W. G., Levrero M., Wang J. Y. (1999). The tyrosine kinase c-Abl regulates p73 in apoptotic response to cisplatin-induced DNA damage.. Nature.

[PDF_00391] Ichimiya S., Nimura Y., Kageyama H., Takada N., Sunahara M., Shishikura T., Nakamura Y., Sakiyama S., Seki N., Ohira M. (1999). p73 at chromosome 1p36.3 is lost in advanced stage neuroblastoma but its mutation is infrequent.. Oncogene.

[PDF_00395] Jost C. A., Marin M. C., Kaelin W. G. (1997). p73 is a simian [correction of human] p53-related protein that can induce apoptosis.. Nature.

[PDF_00397] Kaghad M., Bonnet H., Yang A., Creancier L., Biscan J. C., Valent A., Minty A., Chalon P., Lelias J. M., Dumont X. (1997). Monoallelically expressed gene related to p53 at 1p36, a region frequently deleted in neuroblastoma and other human cancers.. Cell.

[PDF_00401] Lagergren J., Bergström R., Lindgren A., Nyrén O. (1999). Symptomatic gastroesophageal reflux as a risk factor for esophageal adenocarcinoma.. N Engl J Med.

[PDF_00404] Levine A. J. (1997). p53, the cellular gatekeeper for growth and division.. Cell.

[PDF_00406] Mai M., Qian C., Yokomizo A., Tindall D. J., Bostwick D., Polychronakos C., Smith D. I., Liu W. (1998). Loss of imprinting and allele switching of p73 in renal cell carcinoma.. Oncogene.

[PDF_00409] Mai M., Yokomizo A., Qian C., Yang P., Tindall D. J., Smith D. I., Liu W. (1998). Activation of p73 silent allele in lung cancer.. Cancer Res.

[PDF_00411] Montesano R., Hollstein M., Hainaut P. (1996). Genetic alterations in esophageal cancer and their relevance to etiology and pathogenesis: a review.. Int J Cancer.

[PDF_00414] Nimura Y., Mihara M., Ichimiya S., Sakiyama S., Seki N., Ohira M., Nomura N., Fujimori M., Adachi W., Amano J. (1998). p73, a gene related to p53, is not mutated in esophageal carcinomas.. Int J Cancer.

[PDF_00418] Nomoto S., Haruki N., Kondo M., Konishi H., Takahashi T., Takahashi T., Takahashi T. (1998). Search for mutations and examination of allelic expression imbalance of the p73 gene at 1p36.33 in human lung cancers.. Cancer Res.

[PDF_00424] Takahashi H., Ichimiya S., Nimura Y., Watanabe M., Furusato M., Wakui S., Yatani R., Aizawa S., Nakagawara A. (1998). Mutation, allelotyping, and transcription analyses of the p73 gene in prostatic carcinoma.. Cancer Res.

[PDF_00427] Walsh T. N., Noonan N., Hollywood D., Kelly A., Keeling N., Hennessy T. P. (1996). A comparison of multimodal therapy and surgery for esophageal adenocarcinoma.. N Engl J Med.

[PDF_00436] Yokomizo A., Mai M., Tindall D. J., Cheng L., Bostwick D. G., Naito S., Smith D. I., Liu W. (1999). Overexpression of the wild type p73 gene in human bladder cancer.. Oncogene.

[PDF_00439] Zhu J., Jiang J., Zhou W., Chen X. (1998). The potential tumor suppressor p73 differentially regulates cellular p53 target genes.. Cancer Res.

